# How might global health master deadly sins and strive for greater virtues?

**DOI:** 10.3402/gha.v7.23411

**Published:** 2014-03-28

**Authors:** Catherine Panter-Brick, Mark Eggerman, Mark Tomlinson

**Affiliations:** 1 Department of Anthropology, Yale University, New Haven, CT, USA; 2 MacMillan Center for International and Area Studies, Yale University, New Haven, CT, USA; 3 Department of Psychology, Stellenbosch University, Stellenbosch, South Africa

**Keywords:** equity, scale-up, leadership, health systems, global health, interventions

## Abstract

In the spirit of critical reflection, we examine how the field of global health might surmount current challenges and prioritize its ethical mandate, namely to achieve, for all people, equity in health. We use the parlance of mastering deadly sins and striving for greater virtues in an effort to review what is needed to transform global health action. Global health falls prey to four main temptations: coveting silo gains, lusting for technological solutions, leaving broad promises largely unfulfilled, and boasting of narrow successes. This necessitates a change of heart: to keep faith with the promise it made, global health requires a realignment of core values and a sharper focus on the primacy of relationships with the communities it serves. Based on the literature to date, we highlight six steps to re-orienting global health action. Articulating a coherent global health agenda will come from principled action, enacted through courage and prudence in decision-making to foster people-centered systems of care over the entire lifespan.

Global health has experienced the ‘best of times’ over the course of the past two decades. Developmental Assistance for Health (DAH) grew from about $5.7 billion in 1990 to $10.8 billion in 2001, before almost tripling to $28.2 billion in 2012 (1). There have been transformative successes such as a reduction in child under-5 mortality, from 11.97 million in 1990 to 6.91 million in 2011 (2). Yet this field faces numerous challenges: poor global governance, fragmented leadership, and poor priority-setting processes (1), as well as the relative inattention to the need for systematic critical reflection (3). Linked to these difficulties is a skewed resource allocation of global health funding, relative to the global disease burden. Thus HIV/AIDS, malaria, and tuberculosis are the ‘big three’ receiving 80% of global research and development funding, while pneumonia, meningitis, and diarrheal diseases receive ‘table crumbs’ despite contributing 25% more to the global burden of disease than the ‘big three’ combined (4).

We offer a commentary regarding what it would take to surmount these challenges in order to shift some of the predominant agendas that characterize the field of global health. In the spirit of critical reflection, we structure this paper in terms of learning from past mistakes and prioritizing core values – using the ethical parlance of mastering deadly sins, and striving for greater virtues. Structurally, global health has broken faith with its core ethical mandate of addressing the root causes of poor health outcomes, falling prey to four main temptations – coveting silo gains, lusting for technological solutions, leaving broad promises largely unfulfilled, and boasting of narrow successes. These are capital sins in the sense that they engender serious misdeeds and careless misdemeanors, and necessitate a change of heart. A sharper focus on values and dispositions – aligned with cardinal virtues of justice, courage, prudence and restraint – is needed to transform global health action.

## The ‘sins and virtues’ analogy

**Fig. 1:**
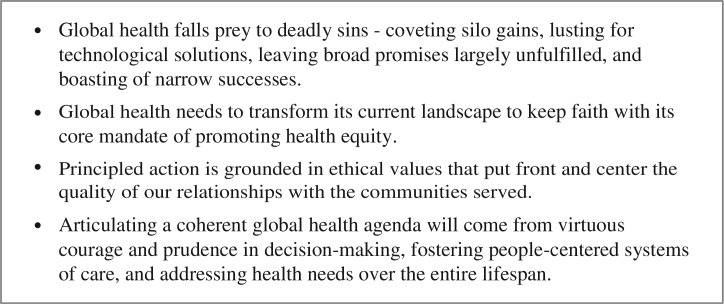
How sins and virtues articulate themselves in global health agendas.

While deadly sins are those actions and omissions that ‘break faith’ with one's relationship with God, cardinal virtues are moral and intellectual habits that dispose us to reason well, informing the ‘first principles’ of a power to act. How do sins and virtues manifest themselves in global health (Fig. 1)? By analogy with theological reasoning, deadly sins are *modus operandi* that break faith with a promise made, namely to address the persistence of harmful and unfair health outcomes. This ‘breaking of faith’ demands a realignment of core values and a sharper focus on the primacy of relationships with the communities we serve. The field of global health has reached a ‘critical juncture’ (5). While its moral, analytical, and operational foundations are clear- a commitment to achieve health equity, by addressing the root causes of unnecessary ill-health (5, 6) - the relational foundation of this commitment is often short-changed. Global health action has readily operated within a harm-reduction paradigm that is necessary but not sufficient to foster human wellbeing. To shift the rather ‘thin’ ethical and social commitment of this paradigm, Gunderson and Cochrane (7) advocated a deeper accountability, one that puts front and center the relationships, values, and structures conducive to the flourishing of human lives. How do we show, for instance, ‘deep accountability’ to fellow human beings to foster wellness, dignity, and capabilities, over and beyond efforts to tackle disease, misery, and pathologies during the span of human existence? Our ‘sins and virtues’ analogy thus serves to highlight both moral and relational issues – a commitment to values and accountability to human beings - in global health's efforts to ‘keep faith’ with a higher purpose, namely to promote fairness and wellness.

## Coveting silo gains

Political priorities for ‘vertical approaches’ to many global health interventions mirror the fragmented and often conflicting nature of the agencies tasked with improving health – a silo mentality that covets silo gains. In the parlance of deadly sins, the fragmented leadership of silo mentalities denotes *avaritia* and *invidia*, namely covetousness and insatiable desire in the pursuit of gains. Thus, greed and envy destroy the impetus for strategic partnerships in global health, encouraging excessive competition. For example, Shiffman has shown how organizational rivalries, poor leadership, gender inequalities, and the lack of a resonating frame significantly impeded the ability of stakeholders and agencies to achieve the rapid reductions in maternal mortality that were seen in the area of neonatal mortality (8, 9). Linked to this is the vast array of global agencies active in international health, estimated by McColl to comprise some 26 UN agencies, 20 global and regional funds, 40 bilateral donors, and 90 global health initiatives (10). The result is a confusing playing field, leading to unnecessary replication of bureaucracies or large gaps of services across agencies.

The *capital sin* identified here is that stakeholders are motivated to ‘follow the money’, subverting medical and public health practice, academic research agendas, incentives for intervention, and selection of markets (11). Competition may be important for excellence, but there is evidence that global health initiatives can negatively impact on country priorities, leading to agendas based on who can leverage issues for silo institutional gain, rather than which health issue is most likely to produce sustained local benefit.

## Lusting for magic bullet solutions

Why is the panacea of many global health programs focused on ‘magic bullet’ solutions to complex health problems and the proliferation of short-term approaches, rather than synergistic and life-course approaches to global health interventions (12)? Magic bullet interventions are prone to a toxic mix of *ira* and *luxuria* – impatience with the many obstacles to disease eradication and lust for technological fame (13). Many global health programs based on magic bullets (whether a vaccine for HIV, a micronutrient supplement for malnutrition, or a tablet for neglected tropical diseases) have shown limited success or poor diffusion (14). The fact that 50 years after the development of the measles vaccine, coverage in African countries such as Uganda is as low as 55%, and below 70% in countries such as Congo, Liberia, and Mozambique (15), is testament to how the reality of ‘real world’ delivery subverts biomedical lust for the power of ‘surgical intervention’. Controversially, Birn has argued that a focus on disease (rather than health) and on technical (rather than social) solutions emerged out of financial and private sector involvement in the 1980s that reigned in public health activism, in favor of narrower mandates (16).

## Leaving broad promises unfulfilled

Sloth (*acedia*) in tackling unfulfilled promises is another capital sin, with lazy thinking responsible for finding oneself remaining well short of the finish line. One example is newborn screening programs in the United States, which flag up the risks of rare metabolic conditions but are not structurally designed to provide follow-up care for parents of newborns at-risk (11). In offering a service with targeted benefits but insufficiently tangible health care provision, such programs fall short of all expectations. Another example of unfulfilled expectations is the implementation of community health worker (CHW) programs, hindered by barriers to effective scale-up, with large-scale programs undermined by high attrition and low performance. Health care systems deploying CHWs have achieved many successes (17, 18) but have also been characterized by inconsistent supervision, inadequate training, and ineffective linkages to the health system (19, 20). Large gaps remain between small-scale efficacy studies and large-scale interventions, demanding effective strategies to support the management and supervision of CHWs in order to ensure quality of implementation (21).

Similarly, delivering efficacious treatments under ideal conditions is quite different from implementation at scale. Community-based interventions are often embedded in dysfunctional health systems, within the messiness of family life, the stress engendered by poverty or violence, the competing interests of multiple stakeholders, and the sharp bite of unintended consequences to good intentions. Thus, lazy thinking characterizes many global health initiatives that leave unaddressed large gaps, between promises of health promotion and actual delivery of effective care.

## Boasting of narrow successes

Global health policy tends to narrowly replicate its past successes – for example, in targeting health beliefs without targeting the structural drivers of ill-health. It often lacks a coherent theory of behavior change, one that is reliably used to develop, implement, and test strategies of health prevention. Of course, there have been many remarkable successes in global health; a case in point is tobacco control, described as perhaps the most rational and evidence-based policy that exists in health care (22), with the Framework Convention on Tobacco Control (23) providing an exemplar of the power of structural interventions over narrowly focused behavioral interventions. Too often, however, the need for combined action at all levels – structural, sociocultural, familial, and individual – remains a global health imperative stymied by the lack of strategic integration. We know that interventions narrowly predicated on a health belief model, which do not bring about changes in the social and economic fabric of society, often fail to produce lasting behavior change and equitable health gains (24, 25). In marshaling research evidence to inform interventions, global health falls prey to the deadly sin of pride: *superbia*, a love of self, and vainglorious satisfaction with successes achieved thus far.

The lesson here is to fight a tendency to be incommensurately proud of short-term successes in narrowly defined outcomes. Worldwide, impressive reductions in child and neonatal mortality rates have been achieved. However, in their provocative critique of global health efforts, Nichter and Cartwright argued that the huge successes in reducing child mortality had, in narrowly focusing on child survival, all but saved the next generation of smokers for the tobacco industry (26). The global strategy for the prevention of non-communicable diseases (27) is a good example of policy that requires tackling multinational interests regarding what we eat (28), how little we do, the amount of alcohol and tobacco we consume, and what medicines we can afford, along with sophisticated programs of behavior change. It requires a broad and coherent agenda that advocates and legislates for effective behavioral, cultural, and political changes.

## Transformative virtues

We advocate a sharper focus on core values and deep accountability, an approach that may resonate with the goals of an upcoming generation of global health researchers and practitioners. Global health has already crystallized the moral, social, economic, and political argument linking social equity with health equity, championing the cause of greater social *justice* in matters of health (29). A case in point is the landmark Commission on the Social Determinants of Health (30) that foregrounded social inequities at the core of so many biological health issues. Likewise, initiatives such as the Movement for Global Mental Health and the Lancet Series on mental health (31, 32) have effectively mobilized a call for action based on the principles of human rights, equity, and disease burden (33).

How might global health transform its current landscape to keep faith with its core mandate? Sins are not just mastered simply through resolution but through a transformative ‘vision’. We need *courage* to rise above coveting silo gains, sluggishly addressing unfinished agendas, boasting of short-term successes, or just following the money. Moving forward, the ability to articulate a more coherent global health agenda will come from striving for greater virtues, to realize a fuller transformative vision in the pursuit of health equity as expressed in all dimensions of physical, social, and emotional wellbeing. Among the chief virtues underscoring this commitment are justice and courage, but also virtuous *prudence* and *restraint* in decision-making. Certainly, greater discernment of judgment is needed with respect to weighing the intended and unintended consequences of social action, including medical or public health interventions (3). The lesson is only just being learned in the field of neglected tropical diseases, with warnings that mass drug administration programs have serious side-effects when delivered to populations who are undernourished, which raises concerns with program implementation without due monitoring or due integration with existing health care delivery systems (34). Cardinal virtues are, in essence, moral and relational dispositions that orient principled action: espousing the virtues of courage and prudence, as well as advocating justice, would help conquer deadly temptations and give greater coherence to global health agendas.

## Next steps to re-orient global health action

Current goals negotiated for global health build upon a tremendous legacy: the 1978 Declaration of Alma Ata, the 1993 World Bank Development Report, the 2000 UN Declaration for achieving the Millennium Development Goals, and the 2008 WHO Commission on the Social Determinants of Health. For instance, the Global Health 2035 framework (35) provides a new investment framework to achieve dramatic health gains within the timeline of just one generation. Strengthening international and national leadership is an important answer to the ‘how’ question we face for implementing this roadmap. There is an important case to be made for strengthening the leadership and functioning of agencies such as the WHO and World Bank, rather than creating new funds and initiatives in the spirit of targeting specific goals (36). A new architecture of global health governance is required to support a model of ‘collaborative care’ in primary health care settings, to integrate, for example, mental health care with the management of non-communicable diseases (37).

Our systems of health care delivery will best follow a people-centered, rather than a technology-centered agenda. While the quest for new vaccines and microbicides must undoubtedly continue, the search for singular medicalized solutions must be consigned to a supporting actor role on the global health stage. Efforts to ‘reimagine global health’ (38) give specific attention to local lives and the context of local institutions that shape priorities for wellbeing and constrain agency (39). We need an ecological approach that is implemented across the lifespan and works on multiple levels, from the individual to the structural, the biological to the cultural (12). Kim and colleagues recently proposed a framework for global health delivery (40), which acknowledges the complexity of system analysis and proposes a way forward, namely integrating vertical interventions into a shared delivery infrastructure, in order to reap the benefits of scale (40). They outlined the processes at both micro-level (e.g. water and sanitation) as well as macro-level (e.g. housing and employment) that will be crucial for ensuring future successes in global health. Health systems strengthening, with ‘diagonal’ interventions to bridge technological innovations with institutional leadership, provides a crucial opportunity for global health action (41).

## Conclusions

In brief, current literature has highlighted at least six ways to re-orient global health action. Specific future steps are to strengthen institutional leadership; follow a people-centred and life-course agenda; theorize global health in a manner which robustly integrates structural and behavioral change in systems of care; espouse a coherent strategic frame for financial incentives and effective leadership; deliver with more consistency on medical and public health promises; and listen more carefully to what locally matters in everyday life.

In research and practice, global health creates significant opportunities to engender a better world. In this commentary, we used the analogy of ‘sins and virtues’ to highlight how global health might re-orient itself to better ‘keep faith’ with a promise to foster wellness and equity in people’s lives. To act in a principled way, global health does need to focus on ethics and accountability. Having successfully advocated greater social justice in matters of health, it remains to show enhanced ‘courage’ to deliver with more consistency on medical and public health undertakings, and ‘prudence’ in decision-making to listen more carefully to what locally matters for people in local contexts. Articulating a more coherent global health agenda will come from enabling people-centered systems of care to address physical, social, and mental wellbeing over the entire life span.
